# Within-family analysis of PRS_313_: insights into breast cancer risk prediction

**DOI:** 10.1016/j.jgeb.2025.100605

**Published:** 2025-10-25

**Authors:** Hossein Lanjanian, Sahand Tehrani Fateh, Mahdi Akbarzadeh, Maryam Moazzam-Jazi, Maryam Zarkesh, Sajedeh Masjoudi, Asiyeh Sadat Zahedi, Leila Najd-Hassan-Bonab, Sara Asgarian, Mohammad Reza Moghaddas, Kamran Guity, Bita Shalbafan, Amirabbas Momenan, Davood Khalili, Fahimeh Ramezani Tehrani, Mehdi Hedayati, Fereidoun Azizi, Maryam S Daneshpour

**Affiliations:** a Cellular and Molecular Endocrine Research Center, Research Institute for Endocrine Molecular Biology, Research Institute for Endocrine Sciences, Shahid Beheshti University of Medical Sciences, Tehran, Iran; b School of Medicine, Tehran University of Medical Sciences, Tehran, Iran; c University of Iceland School of Health Sciences, Faculty of Medicine, Reykjavik, Iceland; d Clinical Research Development Center of Labbafinejad Hospital, Shahid Beheshti University of Medical Sciences, Tehran, Iran; e Prevention of Metabolic Disorders Research Center, Research Institute for Metabolic and Obesity Disorders, Research Institute for Endocrine Sciences, Shahid Beheshti University of Medical Sciences, Tehran, Iran; f Reproductive Endocrinology Research Center, Research Institute for Endocrine Molecular Biology, Research Institute for Endocrine Sciences, Shahid Beheshti University of Medical Sciences, Tehran, Iran; g Endocrine Research Center, Research Institute for Endocrine Disorders, Research Institute for Endocrine Sciences, Shahid Beheshti University of Medical Sciences, Tehran, Iran

**Keywords:** Breast Cancer, Within-Family Analysis, Tehran Cardiometabolic Genetic Study (TCGS), Population-Specific PRS Models, Middle Eastern Population, Breast Cancer Genetics, Monogenic Variants

## Abstract

Polygenic risk scores (PRS) incorporate numerous genetic variants, each with a small impact on cancer pathogenesis, to provide personalized risk assessments. This study investigated, the applicability of PRS_313_ for breast cancer risk both in the general population and within families (patients vs different groups of their relatives) from the Tehran Cardiometabolic Genetic Study (TCGS)cohort. The cohort included 72 breast cancer cases and 2,603 controls. PRS_313_ showed no significant difference between cases and controls, and logistic regression indicated no significant association between PRS_313_ and breast cancer risk (OR: 1.24, 95 % CI: 1.002–1.54). However, PRS differences between patients and their first- and second-degree relatives showed strong statistical significance. The monogenic variant analysis identified 21 loss-of-function (LOF) variants in the cohort, but only one (BRCA2 9976A > T) was common among breast cancer cases. Our findings highlight the importance of within-family analysis of PRS and the challenges of applying European-derived PRS models to diverse populations.

## Highlights

• First Study Evaluating PRS313 in an Iranian Population
• Within-Family PRS Comparison
• Lack of Significant Association in General Population
• Significant PRS Differences in Family Groups
• Importance of Population-Specific PRS Models

## Graphical abstract

**Figure:**
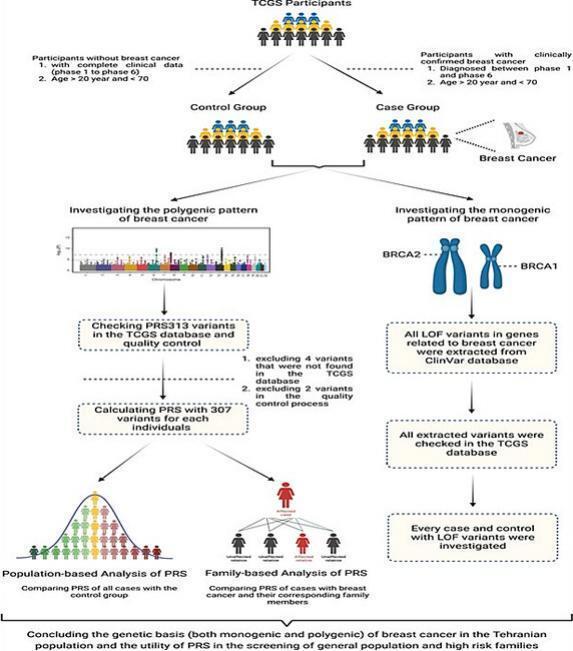

## 1 Introduction

Breast cancer is the most prevalent cancer among women worldwide, presenting a significant public health challenge. While environmental and lifestyle factors contribute to breast cancer risk, genetic susceptibility plays a crucial role, particularly in early-onset and familial cases.^1^ Traditional genetic screening often focuses on rare, high-penetrance mutations such as BRCA1, BRCA2, Epigenetic,^2^, ^3^ and Non-Coding RNAs;^4^ however, these account for only a small fraction of cases. Increasing evidence indicates that common low-risk variants can provide valuable insights into individual susceptibility when collectively considered through polygenic risk scores (PRS). Extensive studies have examined the complex relationship between genetic predisposition and environmental factors in the development of breast cancer. Additionally, breast cancer is the most common cancer among women in Iran, representing a significant public health challenge and imposing a considerable burden on the healthcare system. According to a recent *meta*-analysis, the age-standardized incidence rate of breast cancer in Iran is estimated at 32.1 per 100,000 women. The high prevalence and incidence of breast cancer and its high mortality and morbidity underscores the urgent need for effective strategies for early detection and risk stratification.^5^, ^6^

Although the importance of family history as a major risk factor has been recognized for a long time, current progress in genomics has revealed the possibility of polygenic risk scores (PRS) to enhance risk assessment evenmore.^7^ Notably, PRS and family history often provide complementary rather than overlapping information, thereby improving the overall accuracy of risk prediction models.^8^, ^9^, ^10^, ^11^ Polygenic risk scores summarize the aggregate effects of multiple common genetic variants, each contributing a small effect to disease risk.^12^, ^13^, ^14^ The PRS_313_ model, developed from genome-wide association studies (GWAS), integrates 313 single nucleotide polymorphisms (SNPs) associated with breast cancer and has demonstrated predictive utility across diverse populations. Despite growing interest in PRS, most validation studies have focused on population-based case-control designs. These approaches may be susceptible to confounding from population stratification and environmental heterogeneity. The PRS_313_ is the most extensively studied and well-established polygenic risk score for breast cancer, incorporating 313 common genetic variants associated with disease susceptibility. It has demonstrated robust predictive performance in populations of European ancestry and is increasingly integrated into risk stratification models alongside traditional clinical and lifestyle factors. Although PRS has the potential to improve risk prediction, there are still challenges in effectively combining PRS and family history in healthcare decision-making, as their effectiveness differs among various populations and ancestries.^15^, ^16^, ^17^, ^18^ On the other hand, Within-family studies, which compare affected individuals to their close relatives, offer a complementary framework by inherently controlling for shared ancestry and familial environment. This design enhances the ability to detect genetic associations by reducing confounding and has been underutilized in PRS evaluations.

In this study, we assess the predictive performance of the breast cancer PRS_313_ panel in both a population-based and within-family context using data from the Tehran Cardiometabolic Genetics Study (TCGS). By leveraging the distinct features of the TCGS cohort—including its large sample size, family-based design, and representative Iranian population—we aim to evaluate the accuracy of PRS_313_ in identifying individuals at increased risk for breast cancer. Specifically, we examine the association between PRS_313_ and breast cancer risk, as well as its distribution among affected individuals and their unaffected female relatives.

Our retrospective cohort design incorporates a robust within-family validation framework to disentangle breast cancer risk's genetic and environmental components. By comparing PRS-derived risk estimates with actual disease outcomes across related individuals, we aim to clarify the independent and joint contributions of polygenic risk and family history. This dual analytical approach not only tests the robustness of PRS_313_ but also informs its potential clinical utility in personalized risk assessment and targeted prevention strategies for breast cancer^.^^19^

## 2 Method

### 2.1 Study population

This study leverages data from the TCGS, a 25-year-old large-scale family-based longitudinal cohort study. TCGS represents the Iranian population, encompassing all Iranian ethnicities as well as long-term residents (≥25 years), with follow-ups scheduled every 3 years ^19^ Therefore, it is an unselected cohort, eliminating concerns about clinical-referral ascertainment bias. Since TCGS is a cohort of the normal population without any clinical intervention or selection, it offers a unique opportunity to study penetrance without bias related to clinical referral. Ethical approval for the TCGS project was obtained from the Research Institute for Endocrine Sciences, Shahid Beheshti University of Medical Sciences (code: “IR.SBMU.ENDOCRINE.REC.1395.366″), adhering to the principles of the Helsinki Declaration and its subsequent amendments. Written informed consent was obtained from all participants.

### 2.2 Phenotyping

Our study focuses on two key risk factors: age and family history of breast cancer. Participants diagnosed with breast cancer were identified using the following ICD-11 codes: 2C61.0 (Invasive ductal carcinoma of the breast); 2C61.1 (Invasive lobular carcinoma of the breast); 2C61.3 (Invasive carcinoma of the breast with mixed ductal and lobular features); 2C61.4 (Invasive carcinoma of the breast, unidentifiable type); 2C6Z (Malignant neoplasms of breast, unspecified); 2E65.2 (Ductal carcinoma in situ of the breast).

Clinicians confirmed breast cancer diagnosis by the use of mammography and pathology results. Because of the constraints in the data at our disposal, we were unable to categorize cases according to their estrogen receptor (ER) and progesterone receptor (PR) status.

Exclusion criteria for this study were as follows: (i) male participants, (ii) individuals with incomplete genetic or phenotypic information, (iii) any personal or family history of breast cancer for controls, and (iv) to minimize potential bias, only one member per family was selected for the control group. Since this study was nested within the framework of the TCGS cohort, the sample size was determined by the availability of eligible breast cancer cases and matched controls. Out of 10,558 female participants in the cohort, 128 were diagnosed with breast cancer and served as the primary case group.

### 2.3 Genotyping

Quality control procedures were conducted using Python, PLINK (V1.07),^20^ and R software (V4.1.0).^21^ DNA samples from TCGS participants were extracted from white blood cells using a standard Proteinase K salting-out procedure.^22^ Following quality checks by electrophoresis and spectrophotometry, the samples were genotyped with HumanOmniExpress-24-v1-0 bead chips (containing 649,932 SNP loci with an average mean distance of 4 kb) at deCODE genetics company (Iceland), according to the manufacturer's recommendations (Illumina Inc, San Diego, CA, USA).^23^ From the total participant pool, 1,500 samples were chosen for whole genome sequencing using the HiSeq X Ten (Illumina) platform, achieving a minimum average coverage of 30 × . Additionally, the un-genotyped variants in the SNP array were imputed using the available whole-genome sequencing data.

Quality control analysis was performed to produce high-quality variants. The methods for whole genome sequencing and quality control have been previously detailed. A combined variant call file (gVCF) was generated for all study subjects, encompassing all genetic variations identified in the TCGS study. These variants were annotated using the most recent version of the Variant Effect Predictor (VEP, ver 105). 19, 23

### 2.4 Monogenic variant analysis

For checking the presence of monogenic variants with rare frequency in cases, we selected the 41 candidate causal genes for breast cancer from the Curated Breast Cancer Genes (CBCG) database (https://cbcg.dk/index.html). Among them, 24 genes had at least one loss of function variant in TCGS. Pathogenic variant information for “Breast Cancer” was also retrieved from the ClinVar database. The information on genetic variants from these 2 databases was compared and merged. The shared variants in TCGS were coded as pathogenic variants concerning monogenic breast cancer in cases. All markers were evaluated for all people with calculated PRS, and the heterozygous and homozygous variants were labeled in this group.

### 2.5 Statistical analysis

#### 2.5.1 Descriptive statistics

Descriptive statistics were calculated for age and other relevant clinical characteristics for both cases and controls. Breast cancer prevalence was determined by dividing the total number of diagnosed female breast cancer cases by the number of female participants aged 20 years and older in the TCGS study.

#### 2.5.2 PRS calculation and comparison

PRS was calculated for each participant by summing the product of the number of effect alleles and the natural logarithm of the odds ratio (OR) for each variant included in the PRS_313_ model. Density plots were generated to visually compare the PRS distribution between cases and controls. For statistical comparison, PRSs were standardized to z-scores.

### 2.6 Association analysis

The association between PRS and breast cancer risk was assessed using logistic regression, with PRS as the independent variable and breast cancer status as the dependent variable. ORs per standard deviation (SD) increase in PRS were calculated to quantify the strength of the association. A receiver operating characteristic (ROC) curve analysis was performed to evaluate the predictive performance of PRS and age when discriminating between cases and controls.

### 2.7 Within-Family analysis

However, since 1997, there have been studies that focus on the effect of genetics on cancer risk by considering siblings, especially twins.^24^, ^25^ We believe the Within family approach has been underestimated in PRS evaluations. Here, in order to identify direct links between PRS_313_ and the risk variant involved in it and the risk of breast cancer, we assess the effectiveness of PRS within families. By creating pairs of patient-relative groups, we account for genetic relatedness and shared environmental factors, which is essential when analyzing heritable traits like PRS. This design helps control for confounding variables that could arise in unrelated individuals. We compared the PRS of breast cancer patients with those of their non-cancerous female relatives, including total relatives, first- and second-degree relatives, as well as specific groups such as sisters, mothers, and daughters. We created pairs of patient-relative groups, ensuring that for each group of relatives, there was a corresponding group of patients. As a result, each patient was represented in the patient group as many times as they had relatives (e.g., their mother, sisters), which mirrored the paired control group structure. The Wilcoxon signed-rank test was employed to assess differences in PRS between paired individuals, accounting for potential correlations within families.

### 2.8 Statistical software

All statistical analyses were conducted using R software (version 4.1.0) ^21^ implemented in RStudio (version 1.3.1093), and Python. Statistical significance was set at an alpha level of 0.05.

## 3 Result

### 3.1 Participant characteristics

**Fig. 1:**
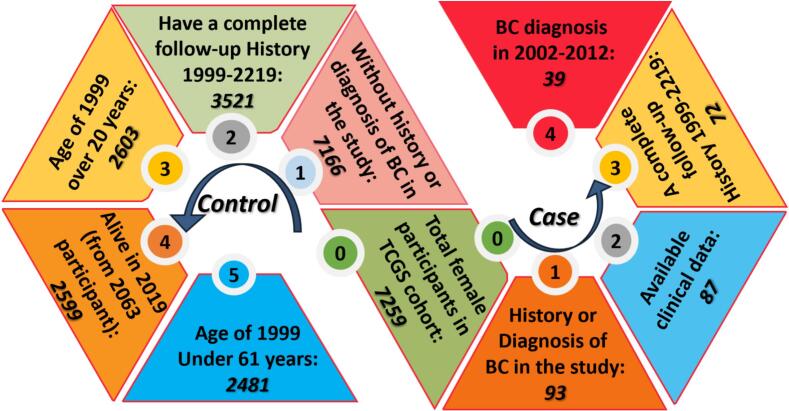
**Participant selection**: The workflow for selecting case and control groups from the TCGS cohorts is illustrated. The cell marked “0″ represents the total number of females (7259) in the cohort (the participant with available genetics data of total 10558). The left loop shows how control group participants were obtained from the cohort population, while the right loop explains the case selection process.

A total of 10,558 female participants were enrolled in the Tehran Lipid and Glucose Study (TLGS), among whom 128 were diagnosed with breast cancer. Of these, 7,259 women had available genetic data and were included in the Tehran Cardiometabolic Genetic Study (TCGS). As illustrated in Fig. 1; after applying the predefined inclusion criteria, 72 breast cancer cases and 2,603 controls were retained for the final analysis. The mean age of cases was 69.18 ± 11.85 years, while that of controls was 62.68 ± 11.85 years (Supplementary Fig. 1). Within the TCGS population, the overall prevalence of breast cancer was 1.22 %. Although the TCGS cohort provided a relatively large pool of controls, the number of breast cancer cases (n = 72) was limited. This imbalance between cases and controls reduced the statistical power to detect modest effect sizes, particularly for polygenic risk score analyses, and should be considered when interpreting the findings.

### 3.2 PRS calculation and distribution

**Fig. 2:**
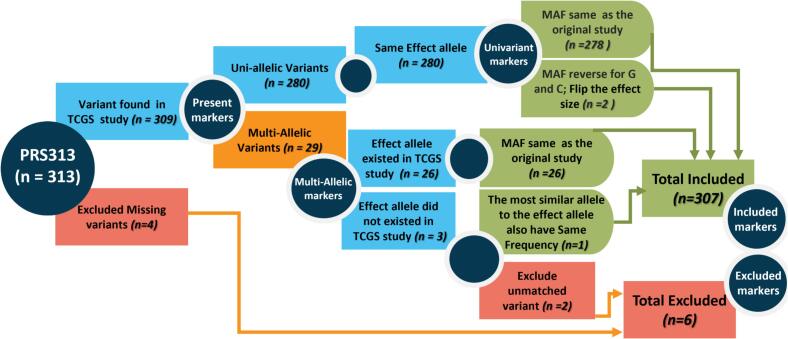
**Markers selection:** The process for filtering markers related to PRS_313_ within the TCGS cohort variants details the steps for examining the presence, frequency, and allelic effect of these markers.

**Fig. 3:**
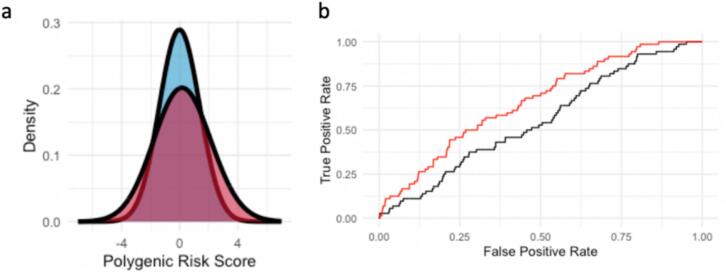
(**a)** The distribution of cases (in red) and controls (in blue) for PRSs obtained by PRS313 (**b)** ROC curve for the performance of PRS313 in discriminating cases from controls. The ROC curve for each PRS is in black and for the model with PRS and age is in red**.** (For interpretation of the references to colour in this figure legend, the reader is referred to the web version of this article.)

Of the 313 SNPs in the PRS_313_ model, 307 were successfully imputed and included in the PRS calculation (Fig. 2, Supplementary Fig. 2, and Supplementary Table 1). The distribution of PRS_313_ scores demonstrated no significant difference between cases and controls (Fig. 3a). The mean PRS_313_ score for cases was −0.14 with a standard deviation of 0.56, while the mean PRS_313_ score for controls was −0.27 with a standard deviation of 0.61.

### 3.3 Association between PRS and breast cancer risk

Logistic regression analysis revealed no significant association between PRS_313_ and breast cancer risk (OR: 1.24, 95 % CI: 1.002–1.54). The area under the receiver operating characteristic (ROC) curve for PRS_313_ was 0.553, indicating poor discriminatory power. In contrast, age demonstrated a moderate predictive ability with an AUC of 0.647. Combining PRS_313_ and age in a multivariable model resulted in a modest improvement in predictive performance (AUC = 0.661), suggesting the limited added value of PRS_313_ beyond age (Fig. 3b).

### 3.4 Within-Family analysis

To investigate the potential role of PRS within families, we compared PRS scores between breast cancer cases and their non-cancerous female relatives. According to Table 1, the PRS of paired groups of patients and their first and second relatives show strong statistical significance. However, the Wilcoxon signed-rank test revealed no significant difference in PRS between paired sisters and mother-daughters, indicating that PRS did not consistently differentiate between cases and their relatives within families (Supplementary Table 2). It is worth to mention that for the paired groups of mother-daughters and daughters-mothers the obtained p-values could be under doubt because of significant age differences. Moreover, there is an insufficient sample size for the sister group.

**Table 1:** This table summarizes the comparative analysis of polygenic risk scores in familial cancer risk assessment. It includes the total number of pairs of cancer cases and their female relatives, along with the W (Wilcoxon-signed-rank sum) test statistic for each subgroup.

Related Group (control)	Sample Size (N)	Age*	W-value	W-Critical-Values; Alpha = 0.05	Z-value	p-value
Total Relatives	200	45.5/48	7946	8444 – 11656	−2.5672	0.0101
First & Second Relatives	125	47.8/46.1	2580	3141 – 4734	−3.3448	0.0008

*Mean Age of onset (Years) of Case/ Mean Age (Years) of related Groups: In the paired groups, each patient was represented in the patient group as many times as they had relatives (e.g., mother, sisters). Consequently, the composition and repetition of cases varied across different relative groups. This variation led to different members and varying repetitions of each member within the corresponding case groups for each relative group.

Calculated by: https://www.socscistatistics.com/tests/signedranks/default2.aspx.

### 3.5 Monogenic variant analysis

**Fig. 4:**
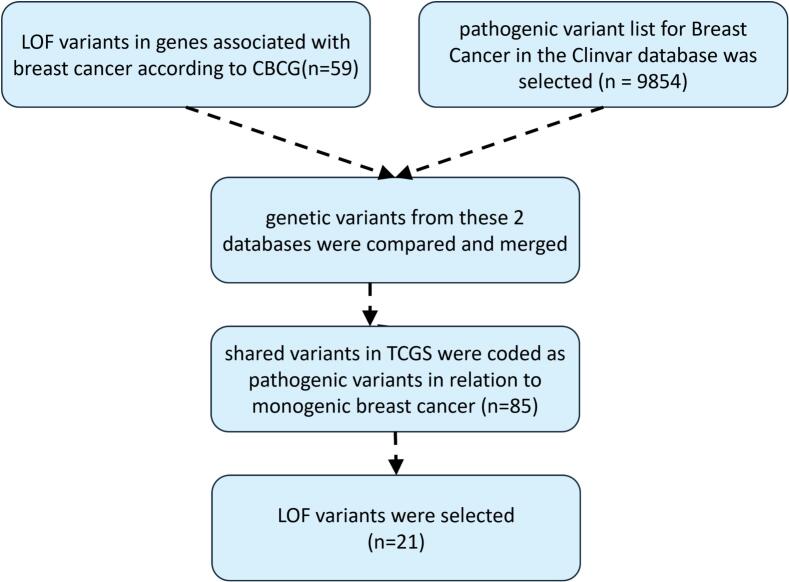
The overview of detecting 21 loss-of-function (LOF) variants in the TCGS cohort.

The analysis of 41 candidate breast cancer genes identified 21 loss-of-function (LOF) variants in the TCGS cohort (Fig. 4 and Supplementary Table 3). The allele frequency of variants varied between 0.00037 and 0.026 among the females investigated in this study. Among 21 LOF variants related to breast cancer, the allele frequency for one variant (BRCA2:c.9976A > T) was common for breast cancer disease in the gnomAD database. Five cases and 65 controls had this variant in the TCGS population. Other 20 variants were not found in the cases. However, 23 controls harbored at least one LOF variant.

## 4 Discussion

**Fig. 5:**
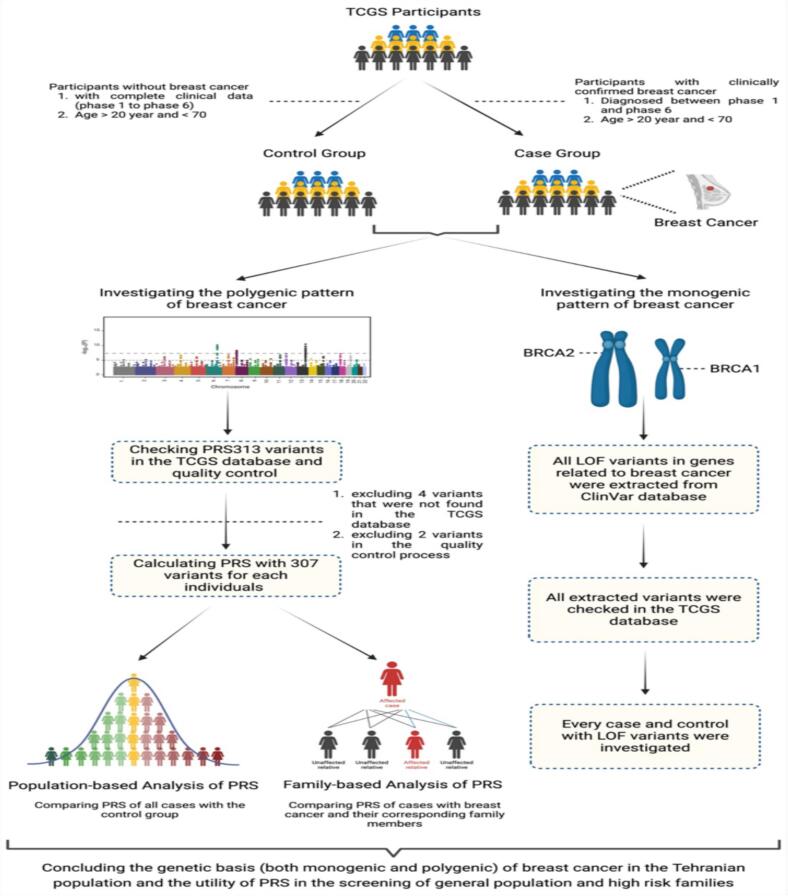
The overview of this study investigating the polygenic and monogenic pattern of breast cancer inheritance.

Our findings suggest the challenges of adapting European-derived PRS to populations with diverse genetic ancestry. The lack of significant association between PRS_313_ and breast cancer risk in our Iranian cohort is consistent with prior research showing the limited generalizability of PRS across varied ethnicities (Fig. 5). The observed discrepancy in PRS performance between European and Middle Eastern populations highlights the importance of developing population-specific PRS models. Performance of PRS_313_ has been evaluated in different ancestries, and generalized quite well in populations with African, Latinx, and Asian ancestry, although with smaller effect sizes and AUCs.^16^, ^17^ Five other PRSs with 45, 34, 75, 77, and 93 markers, respectively, which were developed based on the European population, did not provide a comparable degree of risk stratification for females of African ancestry.^15^ Smaller effect sizes are common in similar cases when assessing the performance of one PRS in a population with different ancestry and are in line with our results about PRS_313_ performance.

Genetic heterogeneity, which refers to genetic variations that are involved in causing the same disease in different populations, is the factor that hinders the generalizability of PRSs in different populations. Not only markers included in PRS are important in discriminating cases and controls, but also, the effect size of each marker is of great importance. Even the mean of PRS distribution has been shown to differ among populations with European and Asian ancestry.^26^ More interestingly, the PRS mean differed in Japanese, Koreans, and Han Chinese, all considered East Asians.^27^ This difference could affect PRS's ability in risk stratifications and underscores the importance of ethnic-specific PRS models.

By focusing on within-family validation, we sought to understand the independent and combined effects of PRS and family history on disease risk prediction, particularly how PRS complements or overlaps with family history in predicting complex traits and diseases. This unique approach enabled us to explore the performance of PRS within families, considering shared genetic and environmental factors. The Wilcoxon signed-rank test found no significant difference in PRS between paired sisters and mother-daughter pairs, indicating that PRS did not reliably differentiate between cases and their relatives within families (see Table 1). Interestingly, nearly all healthy sisters are older than patients (Supplementary file 2). Conversely, there was a strong statistical significance between patients and their first and second-degree relatives. However, the p-values for the mother-daughter and daughter-mother comparisons may be questionable due to significant age differences between these groups. By considering the number of appearances for each patient or relative across different pairs (e.g., a patient’s mother with three daughters and four sisters counts as seven instances), the average onset age of patients was 47.8 years, while the average age of their relatives was 46.1 years (Supplementary file 2). This age difference is not statistically significant, as indicated by the Wilcoxon signed-rank test p-value of 0.20054. These results of extended families are in favor of PRS_313_ in a within-family investigation. Thus, the unexpected result about the sisters’ pairs may be due to the stochastic effect of the small sample size (13 unique patients in 17 sister-sister pairs) that made a weak power for the study of this group.

A study on the Dutch population showed, including the PRS_313_ in family history-based risk prediction, may change screening recommendations in up to 34 % of individuals from families with no pathogenic variants in any of the five breast cancer genes (BRCA1, BRCA1, PALB2, CHEK2, or ATM) modeled in BOADICEA.^28^ In contrast with the results of the previously mentioned study, PRS_313_ could not effectively discriminate between cases and controls in the family history-based risk prediction in our study. This finding suggests that factors beyond genetic predisposition, such as environmental influences or gene-environment interactions, play a crucial role in breast cancer susceptibility within families.

The comparatively low prevalence of breast cancer in our study population may have hindered our ability to identify a substantial association between PRS and breast cancer risk. The lack of comprehensive data on lifestyle factors, hormonal exposures, and other pertinent variables may have impacted the results of the study.

PRS holds great promise for personalized medicine; however, our research underscores the importance of exercising caution when interpreting and implementing PRS findings in clinical settings, particularly in populations with diverse genetic backgrounds. Acknowledging the limitations of currently available PRS models and avoiding overreliance on these tools for risk prediction is crucial.

In order to enhance the practicality of PRS, it is imperative for future studies to concentrate on constructing PRS models that are tailored to various populations with unique characteristics. Moreover, including PRS with other risk factors, such as familial background, clinical and demographic traits, and lifestyle factors, might improve the precision of risk assessment models.

### 4.1 Limitation

A key limitation of our study is the relatively small number of breast cancer cases (n = 72) available in the TCGS cohort, which limits the statistical power to detect modest effects of PRS313. Polygenic risk scores typically capture small-effect variants, and detecting their influence on disease risk often requires large sample sizes, particularly for genome-wide significance. Post-hoc power analysis indicates that, given our sample size and the previously reported effect sizes for PRS313, the study is underpowered to detect associations with confidence, increasing the likelihood of type II errors. Therefore, while our findings provide preliminary insights, larger cohort studies are necessary to robustly evaluate the predictive utility of PRS313 in the Iranian population.

In the within-family analysis, each patient is included multiple times based on their relatives; there might be issues related to over-representation. For example, there are 17 sisters for 13 patients or 200 relatives for 72 patients, thus some patients are included multiple times. The power of the Wilcoxon signed-rank test might be reduced in small samples.

Even though within-family comparisons reduce some confounding variables, unmeasured factors like shared lifestyle or epigenetic modifications within families could still bias the results. Results from within-family comparisons may not generalize well to the broader population, as the genetic architecture might differ between families and populations.

The analysis relies on having a sufficient number of first- and second-degree relatives, which may limit the number of families eligible for inclusion. As in our study, the sister pairs not have enough statistical power.

## 5 Conclusion

Our study highlights the challenges associated with applying European-derived PRS to predict breast cancer risk in an Iranian population. The lack of significant association between PRS_313_ and breast cancer risk, both in the general population and within families, emphasizes the necessity for additional investigation to develop population-specific PRS models. Incorporating additional risk factors and exploring the interplay between genetic and environmental factors are essential for improving breast cancer risk prediction and prevention.

## CRediT authorship contribution statement

**Hossein Lanjanian:** Writing – review & editing, Methodology, Formal analysis, Data curation, Conceptualization. **Sahand Tehrani Fateh:** Writing – original draft, Visualization, Methodology, Formal analysis, Data curation, Conceptualization. **Mahdi Akbarzadeh:** Writing – review & editing, Data curation. **Maryam Moazzam-Jazi:** Writing – review & editing, Data curation. **Maryam Zarkesh:** Writing – review & editing, Data curation. **Sajedeh Masjoudi:** Writing – review & editing, Data curation. **Asiyeh Sadat Zahedi:** Writing – review & editing, Data curation. **Leila Najd-Hassan-Bonab:** Writing – original draft, Data curation. **Sara Asgarian:** Writing – review & editing, Data curation. **Mohammad Reza Moghaddas:** Writing – original draft. **Kamran Guity:** Writing – review & editing, Data curation. **Bita Shalbafan:** Writing – review & editing, Data curation. **Amirabbas Momenan:** Writing – review & editing, Data curation. **Davood Khalili:** Writing – review & editing, Data curation. **Fahimeh Ramezani Tehrani:** Writing – review & editing, Conceptualization. **Mehdi Hedayati:** Writing – review & editing, Data curation. **Fereidoun Azizi:** Writing – review & editing, Data curation. **Maryam S Daneshpour:** Writing – review & editing, Supervision, Formal analysis, Data curation, Conceptualization.

## Funding

This study was funded by the 10.13039/501100007427Research Institute for Endocrine Sciences, Shahid Beheshti University of Medical Sciences (Tehran, Iran), and the scientific and financial support of the deCODE genetic company (Reykjavik, Iceland). This work was supported by the 10.13039/501100003969Ministry of Health and Medical Education (MOHME:700/1736). 10.13039/100020481Shahid Beheshti University for Medical Sciences was granted this project [grant no: 31178]. Iranian molecular medicine network supported the genomic bank.

## Declaration of competing interest

The authors declare that they have no known competing financial interests or personal relationships that could have appeared to influence the work reported in this paper.
