# Supplementary material for: Within-family analysis of PRS313: insights into breast cancer risk prediction
Source: J Genet Eng Biotechnol. 2025 Oct 25;23(4):100605. doi: 10.1016/j.jgeb.2025.100605 (PMC12594898; doi:10.1016/j.jgeb.2025.100605)
Supplement: Supplementary Data 2 [file mmc2.pptx]

## Slide 1
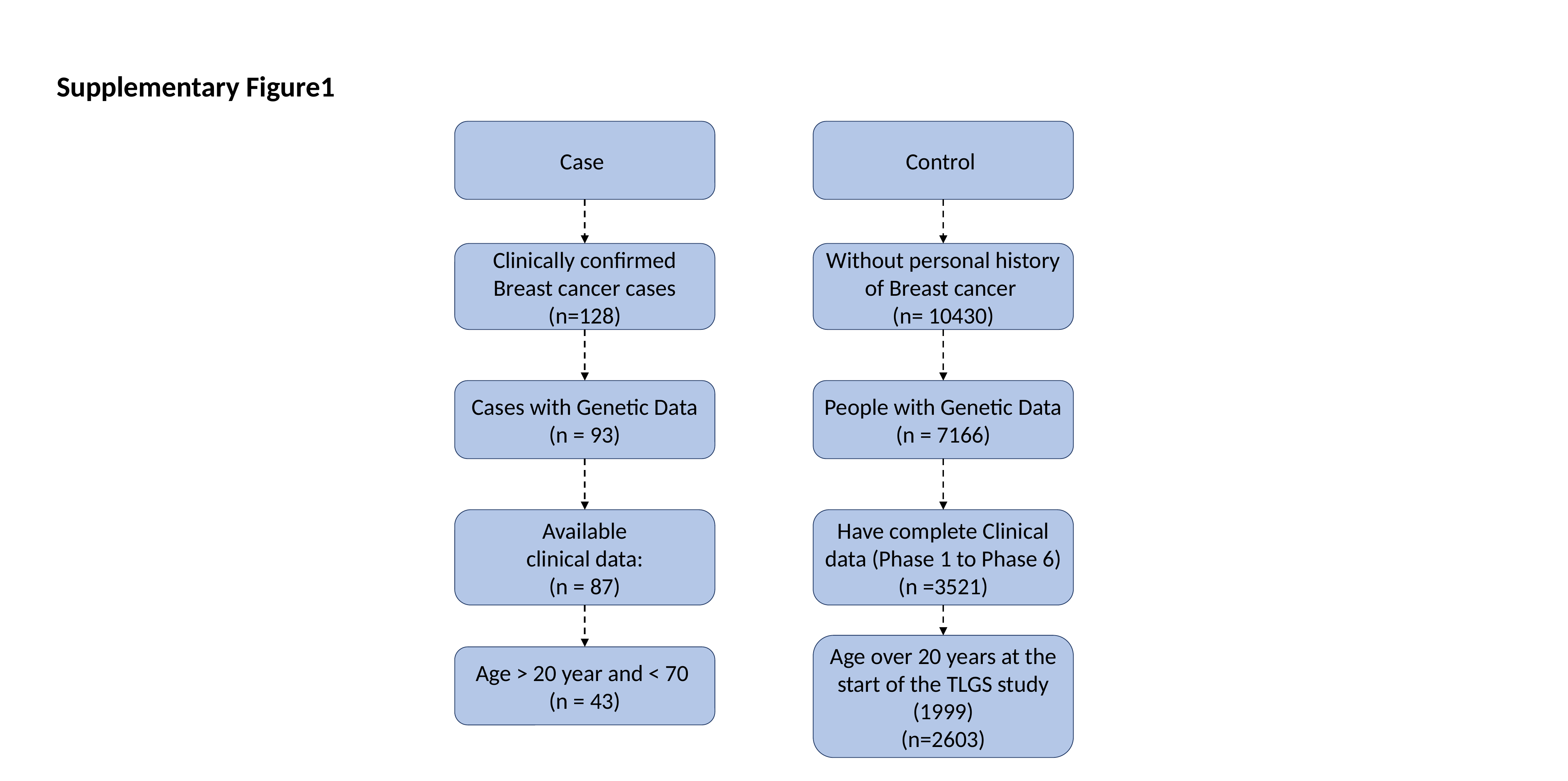

Supplementary Figure1
Case
Control
Clinically confirmed Breast cancer cases
(n=128)
Without personal history of Breast cancer
(n= 10430)
Cases with Genetic Data (n = 93)
People with Genetic Data (n = 7166)
Have complete Clinical data (Phase 1 to Phase 6)
(n =3521)
Available
clinical data:
(n = 87)
Age over 20 years at the start of the TLGS study (1999)
(n=2603)
Age > 20 year and < 70
(n = 43)

## Slide 2
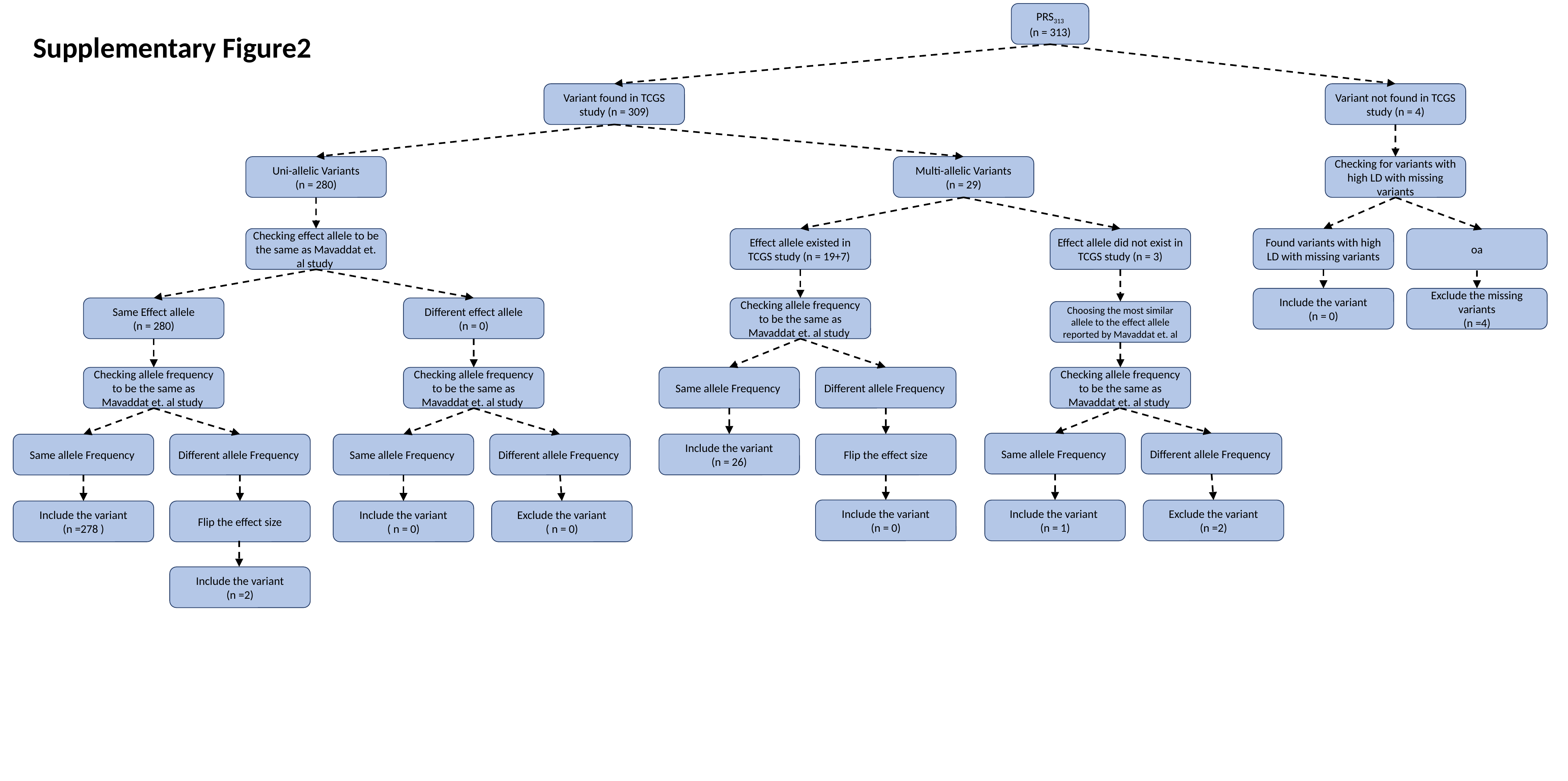

PRS313
(n = 313)
Supplementary Figure2
Variant not found in TCGS study (n = 4)
Variant found in TCGS study (n = 309)
Uni-allelic Variants
(n = 280)
Multi-allelic Variants
(n = 29)
Checking for variants with high LD with missing variants
oa
Found variants with high LD with missing variants
Effect allele existed in TCGS study (n = 19+7)
Effect allele did not exist in TCGS study (n = 3)
Checking effect allele to be the same as Mavaddat et. al study
Include the variant
(n = 0)
Exclude the missing variants
(n =4)
Checking allele frequency to be the same as Mavaddat et. al study
Same Effect allele
(n = 280)
Different effect allele
(n = 0)
Choosing the most similar allele to the effect allele reported by Mavaddat et. al
Different allele Frequency
Same allele Frequency
Checking allele frequency to be the same as Mavaddat et. al study
Checking allele frequency to be the same as Mavaddat et. al study
Checking allele frequency to be the same as Mavaddat et. al study
Different allele Frequency
Same allele Frequency
Include the variant
(n = 26)
Flip the effect size
Different allele Frequency
Different allele Frequency
Same allele Frequency
Same allele Frequency
Include the variant
(n = 0)
Include the variant
(n = 1)
Exclude the variant
(n =2)
Include the variant
(n =278 )
Flip the effect size
Include the variant
( n = 0)
Exclude the variant
( n = 0)
Include the variant
(n =2)
